# Comparative Investigation of the Effects of Adenosine Triphosphate, Melatonin, and Thiamine Pyrophosphate on Amiodarone-Induced Neuropathy and Neuropathic Pain in Male Rats

**DOI:** 10.3390/biomedicines13122965

**Published:** 2025-12-02

**Authors:** Agah Abdullah Kahramanlar, Habip Burak Ozgodek, Ramazan Ince, Bulent Yavuzer, Ozlem Admis, Ali Sefa Mendil, Bilge Ekinci, Halis Suleyman

**Affiliations:** 1 Department of Anesthesiology and Reanimation, Erzurum City Hospital, Erzurum 25070, Turkey; ultradr1@hotmail.com (A.A.K.); brk.ozgodek@gmail.com (H.B.O.); rmzn.ince@hotmail.com (R.I.); 2 Department of Pharmacology, Faculty of Medicine, Erzincan Binali Yıldırım University, Erzincan 24100, Turkey; bulent.yavuzer@erzincan.edu.tr; 3 Biochemistry Laboratory, Mengucek Gazi Education and Research Hospital, Erzincan Binali Yıldırım University, Erzincan 24100, Turkey; drozlem4621@hotmail.com; 4 Department of Pathology, Faculty of Veterinary Medicine, Erciyes University, Kayseri 38039, Turkey; 4022140002@erciyes.edu.tr; 5 Department of Physical Medicine and Rehabilitation, Faculty of Medicine, Erzincan Binali Yıldırım University, Erzincan 24100, Turkey; bilge.ekinci@erzincan.edu.tr

**Keywords:** inflammation mediators, lipid peroxidation, neuroinflammation, neuroprotective agents, oxidative stress, paw withdrawal threshold, peripheral nervous system diseases, Wistar rats

## Abstract

**Background:** Amiodarone is a widely used class III antiarrhythmic agent, but its use can lead to peripheral neuropathy mediated by mitochondrial dysfunction, oxidative stress, and neuroinflammatory injury, while effective preventive options remain limited. Agents that support mitochondrial energy metabolism, sustain redox balance, and modulate inflammation, including adenosine triphosphate (ATP), melatonin, and thiamine pyrophosphate (TPP), may counteract these mechanisms; however, their relative neuroprotective potential in amiodarone-induced neuropathy remains unclear. This study aimed to comparatively evaluate the effects of ATP, melatonin, and TPP on amiodarone-induced peripheral neuropathy and neuropathic pain in rats. **Methods:** Thirty male albino Wistar rats were assigned to five groups: healthy; amiodarone (50 mg/kg/orally); amiodarone + ATP (5 mg/kg/intraperitoneally); amiodarone + melatonin (10 mg/kg/orally); or amiodarone + TPP (20 mg/kg/intraperitoneally). Treatments were given once daily for 14 days. Oxidative stress indices (malondialdehyde (MDA), total glutathione (tGSH), superoxide dismutase (SOD), catalase (CAT)) and proinflammatory cytokines (tumor necrosis factor-alpha (TNF-α), interleukin-1 Beta (IL-1β), interleukin-6 (IL-6)) were quantified in sciatic nerve by Enzyme-Linked Immunosorbent Assay (ELISA). Paw withdrawal thresholds were measured with the Randall-Selitto test before and after treatment. Histopathology was performed using Hematoxylin-eosin staining. **Results:** Amiodarone exposure resulted in pronounced elevations in MDA and proinflammatory cytokine levels, accompanied by significant reductions in tGSH, SOD, CAT activities, and paw withdrawal thresholds. ATP, melatonin and TPP ameliorated these alterations to varying degrees. Among them, TPP provided the most robust antioxidant and anti-inflammatory effects, followed by ATP and melatonin. Histopathological examination confirmed most severe axonal degeneration, interstitial edema and Schwann cell proliferation in the amiodarone group, with substantial amelioration in the TPP-treated rats. **Conclusions:** Amiodarone induces neuropathic pain through oxidative and inflammatory injury to peripheral nerves. TPP exhibited superior neuroprotective efficacy compared with ATP and melatonin, highlighting its potential as a candidate therapeutic agent for amiodarone-related neuropathy. Further clinical research is warranted to support translational application of these findings.

## 1. Introduction

Amiodarone, a benzofuran-derived compound, is classified among class III antiarrhythmic agents and is characterized by its iodine-containing structure [1]. It is widely used in clinical practice for the treatment of atrial fibrillation [2]. Amiodarone is known to enhance hydrogen peroxide synthesis in mitochondria, inhibit complex I activity, and reduce adenosine triphosphate (ATP) levels by inducing uncoupled oxidative phosphorylation [3]. It has been demonstrated in cell culture that amiodarone inhibits mitochondrial complex I activity and ATP biosynthesis, increases reactive oxygen species (ROS), and decreases superoxide dismutase (SOD) and catalase (CAT) activities [4]. Evidence indicates amiodarone decreases cellular respiration and reduces ATP levels in human blood cells in a manner that is both dose and time dependent [5]. It is also known that this drug, which is widely used for arrhythmia, has serious toxicity affecting the liver, heart, lungs, skin, and thyroid [6]. Another complication of amiodarone is peripheral neuropathy, which affects approximately 10% of treated patients [7]. Peripheral neuropathy is a common neurological disorder due to peripheral nerve injury that adversely affects quality of life [8]. Drug-induced peripheral nerve damage is referred to as drug-induced peripheral neuropathy (DIPN) [9]. Studies conducted on DIPN indicate that its direct mechanism involves axonal transport impairment, while its indirect mechanism is linked to oxidative stress and mitochondrial dysfunction [8]. Evidence from studies on amiodarone-induced DIPN reveals demyelination, loss of large axons accompanied by lysosomal inclusions, as well as pronounced oxidative stress and lysosomal damage [9]. It has also been suggested that, in addition to oxidative stress, pro-inflammatory cytokine release plays a role in amiodarone injury [10].

ATP, whose protective effect in amiodarone-induced neuropathic pain we will discuss, is both an energy currency and a signaling molecule. It is synthesized endogenously and can also be taken as an oral supplement [11]. The literature indicates that decreased ATP production may lead to peripheral nerve damage and that mitochondrial dysfunction may play a role in peripheral neuropathies [12]. Mitochondrial damage has been established as one of the key factors in the pathogenesis of neuropathy [13].

Melatonin (N-acetyl-5-methoxytryptamine), whose protective effect we will investigate in amiodarone-induced neuropathy and neuropathic pain, is a circadian rhythm-regulating sleep hormone with physiological and biological roles [14]. Melatonin has antioxidant, ROS scavenging, and anti-inflammatory properties and has the capacity to regulate mitochondrial homeostasis [15,16,17]. Studies have shown that melatonin preserves mitochondrial membrane potential and inhibits oxidative stress-induced dysfunction, particularly in neurodegenerative disease models [18]. Numerous studies indicate that melatonin treatment reduces diabetic or peripheral neuropathies by inhibiting oxidative stress and inflammatory pathways via antioxidant mechanisms [19,20,21]. Melatonin has been proposed to play an important role in reducing neuroinflammation associated with neuropathy-induced depressive and anxiolytic behaviors [22].

Thiamine pyrophosphate (TPP) is the active metabolite of thiamine [23]. TPP, whose importance for neuropathic pain will be evaluated, plays a critical role in the Krebs cycle and cellular metabolism [24]. Many steps are required for ATP production to proceed smoothly; one such step is the pyruvate dehydrogenase complex, which is essential for the conversion of pyruvate to acetyl-CoA [25]. TPP is required as a cofactor for the proper function of this enzyme complex [26]. It has been emphasized that thiamine is ineffective in the treatment of cisplatin-induced peripheral neuropathic pain, whereas TPP is effective, a finding attributed to cisplatin’s inhibition of thiamine conversion to TPP [27].

Information obtained from the literature suggests that ATP, melatonin, and TPP may be beneficial in the treatment of amiodarone-associated neuropathic pain. Moreover, there are no studies in the literature that have specifically investigated the effects of ATP, melatonin, or TPP on amiodarone-related neuropathic pain. The objective of this study is to investigate and comparatively assess the effects of ATP, melatonin, and TPP on amiodarone-induced neuropathy and associated neuropathic pain in a rat model.

## 2. Materials and Methods

### 2.1. Animals

A total of 30 male albino Wistar rats (9–10 weeks old; body weight: 282–297 g) were used in this experimental study. All experimental animals were obtained from the Laboratory Animals Application and Research Center of Erzincan Binali Yıldırım University (Erzincan, Turkey). Rats were randomly divided into five groups with six animals in each, ensuring similar mean body weights among groups. Prior to the initiation of the experiments, all rats underwent an acclimatization period and were housed in standard wire laboratory cages (20 cm height × 35 cm width × 55 cm length; floor area: 1925 cm^2^), with six animals per cage. Throughout this study, animals were maintained under controlled environmental conditions, including a 12 h light/12 h dark photoperiod, constant ambient temperature (22 ± 2 °C), and relative humidity ranging from 30% to 70%. Throughout the experimental period, animals were supplied with standard laboratory chow (Bayramoglu Feed and Flour Industry Inc., Erzurum, Turkey) and tap water, both offered ad libitum. All animal-related experimental procedures were performed in the laboratories of the Experimental Animal Application and Research Center, Erzincan Binali Yıldırım University.

This study was designed and performed in accordance with Directive 2010/63/EU of the European Parliament on the protection of animals used for scientific purposes (Approval ID: 2016-24-199) and complied with the ARRIVE (Animal Research: Reporting of In Vivo Experiments) guidelines [28].

### 2.2. Reagents and Chemicals

All reagents and chemical compounds used in the present study were of analytical grade and were obtained from commercial sources. Thiopental sodium (Pental Sodyum^®^, 0.5 g vial, catalog No.: 8699508270385) was obtained from Menarini Health and Pharmaceuticals Industry Trade Inc. (Istanbul, Turkey). Amiodarone (Cordarone^®^, 200 mg tablet, catalog No.: 8699542010619) was purchased from Sanofi Synthelabo Pharmaceutical Inc. (Istanbul, Turkey). ATP (ATP^®^ 10 mg/mL solution for injection, catalog No.: 4820117741513) was sourced from Zdorovye Narodu Pharmaceutical LLC. (Kharkiv, Ukraine). Melatonin (Melatonina^®^, 5 mg tablet, catalog No.: 5909990831715) was supplied by Lek-am Pharmaceutical Company Ltd. (Zakroczym, Warsaw, Poland). TPP (Cocarboxylase hydrochloride^®^; 50 mg/2 mL injectable formulation, catalog No.: 4820011070436) was provided by BioPharma (Kyiv, Ukraine).

### 2.3. Experimental Design and Randomization

The sample size was established according to the principle of using the minimum number of animals required to obtain reliable and reproducible results, in compliance with the 4R framework (Reduction, Refinement, Replacement, and Responsibility) [29]. Animals exhibiting signs such as abnormal posture, decreased locomotor activity, or injuries resulting from cage-mate aggression were pre-specified as potential exclusion criteria for both the experimental phase and subsequent data analysis. However, none of the animals fulfilled these criteria, and no exclusions were applied. Randomization was performed using a random number table to allocate animals to the experimental groups. To minimize the risk of bias and control for potential confounding variables, both cages and individual animals were assigned numerical codes throughout this study.

### 2.4. Experimental Groups

Animals were randomly allocated into five experimental groups: a healthy control group (HG); a group treated with amiodarone alone (AMDG; 50 mg/kg, oral); a group receiving a combination of amiodarone and ATP (AATPG; 50 mg/kg, oral + 5 mg/kg, intraperitoneal); a group co-treated with amiodarone and melatonin (AMTNG; 50 mg/kg, oral + 10 mg/kg, oral); and a group administered amiodarone together with TPP (ATPPG; 50 mg/kg, oral + 20 mg/kg, intraperitoneal).

### 2.5. Experimental Procedure

First, baseline mechanical paw withdrawal thresholds of all groups were measured using a Basile analgesimeter (Randall–Selitto method) [30]. Subsequently, the animals in the AATPG (*n* = 6), AMTNG (*n* = 6), and ATPPG (*n* = 6) groups received ATP (5 mg/kg, intraperitoneally), melatonin (10 mg/kg, orally), and TPP (20 mg/kg, intraperitoneally), respectively. The dose and administration route of ATP [31], melatonin [32], and TPP [33] were based on previously validated experimental models in which these agents consistently demonstrated antioxidant, anti-inflammatory, and tissue-protective efficacy. Distilled water was administered in equal volumes as a vehicle to the HG (*n* = 6) and AMDG (*n* = 6) groups. One hour following the administration of either the vehicle or the respective pretreatments (ATP, melatonin, or TPP), amiodarone (50 mg/kg) was administered by oral gavage to all groups except the HG group. This sequence was repeated once daily for 14 consecutive days. This dosing regimen was selected based on previous reports demonstrating that once-daily administration of amiodarone at 50 mg/kg for 14 days reliably induces neuropathy [34]. At the end of this period, paw withdrawal thresholds were measured again. Subsequently, the animals were euthanized under thiopental anesthesia (50 mg/kg, intraperitoneally), and their sciatic nerves were carefully dissected and collected. Malondialdehyde (MDA), total glutathione (tGSH), SOD, CAT, tumor necrosis factor-alpha (TNF-α), interleukin-1 beta (IL-1β), and interleukin-6 (IL-6) levels were determined in the nerve tissue. In addition, the tissues were evaluated histopathologically.

### 2.6. Biochemical Analyses

#### 2.6.1. Preparation of Samples

Approximately 30 mg of sciatic nerve tissue was dissected from each rat and briefly rinsed in ice-cold 0.9% sodium chloride solution to remove residual blood and debris. Following weighing, sciatic nerve tissues were sectioned into smaller fragments, promptly immersed in liquid nitrogen to achieve flash-freezing, and subsequently ground into a fine powder using a pre-chilled mortar and pestle. The resulting tissue powder was homogenized in phosphate-buffered saline (PBS, pH 7.4) at a 1:10 (*w*/*v*) ratio. Homogenates were vortex-mixed for 10 s and centrifuged at 10,000× *g* for 20 min at 4 °C. The supernatant fractions were carefully collected and stored at −80 °C for subsequent biochemical assays. To ensure comparability across groups, biochemical data were normalized to total protein content and reported as nmol/mg protein for MDA and tGSH and U/mg protein for SOD and CAT.

#### 2.6.2. Determination of MDA, tGSH, SOD, CAT, and Total Protein Levels in Sciatic Nerve Tissue

The MDA and tGSH levels, as well as SOD activity, in sciatic nerve tissue were determined using rat-specific ELISA kits (catalog No.: 10009055 for MDA; 703002 for tGSH; 706002 for SOD; Cayman Chemical^®^ Co., Ann Arbor, MI, USA) according to the manufacturer’s instructions. CAT activity was assessed following the method described by Goth [35]. Total protein concentrations were quantified by the Bradford assay [36], which is based on the binding of Coomassie Brilliant Blue G-250 (Catalog No.: 115444, Sigma-Aldrich Chemie GmbH, Taufkirchen, Bavaria, Germany) dye to protein molecules. Absorbance was measured spectrophotometrically at 595 nm, and the resulting values were used for normalization of all biochemical data.

#### 2.6.3. Determination of TNF-α, IL-1β, and IL-6 Levels in Sciatic Nerve Tissue

The levels of TNF-α (ng/L; catalog No.: 201-11-0765), IL-1β (pg/L; catalog No.: 201-11-0120), and IL-6 (ng/L; catalog No.: 201-11-0136) in the supernatant fractions obtained from sciatic nerve tissue homogenates were determined using commercially available ELISA kits (SunRed Biotechnology Co.^®^, Shanghai, China) according to the manufacturer’s instructions.

### 2.7. Histopathological Procedures

All sciatic nerve tissue specimens were initially fixed in 10% neutral-buffered formalin to ensure optimal preservation of tissue architecture for subsequent light microscopic analysis. Upon completion of fixation, the tissue samples were immersed and gently rinsed under continuous tap water flow for 24 h while enclosed in cassettes. Tissue dehydration was subsequently performed through a graded series of ethanol solutions (70%, 80%, 90%, and 100%), followed by clearing in xylene and embedding in paraffin blocks. Paraffin-embedded tissue blocks were sectioned into slices approximately 4–5 µm thick, which were subsequently stained with hematoxylin and eosin (H&E) for histopathological evaluation. Serial paraffin sections were obtained from the sciatic nerve tissues of six animals in each experimental group (*n* = 6). From each section, a single central microscopic field was analyzed at 40× magnification, resulting in a total of six representative images being evaluated per group. Photomicrographs were acquired using the Olympus DP2-SAL imaging software (version 3.3.1.198; Olympus Inc.^®^, Tokyo, Japan). Histopathological alterations in sciatic nerve tissue were defined by the presence of axonal degeneration, Schwann cell proliferation, and interstitial edema. Tissue injury was semi-quantitatively graded using a five-point scale: 0 = no detectable damage, 1 = mild, 2 = moderate, 3 = severe, and 4 = most severe (Table 1). All histopathological assessments were carried out by a pathologist who was blinded to the experimental group assignments.

### 2.8. Statistical Analyses

All statistical evaluations of biochemical and histopathological data were carried out using the IBM SPSS Statistics software package for Windows (version 27.0; IBM^®^ Corp., Armonk, NY, USA, 2020). Graphs and visual representations were generated with GraphPad Prism^®^ software (version 8.0.1; GraphPad Software, San Diego, CA, USA, 2018). Biochemical outcomes are presented as the mean ± standard error of the mean (SEM). The normality of data distribution was assessed using the Shapiro–Wilk test, and the equality of variances was evaluated with Levene’s test (Tables S1 and S2). When the assumption of homogeneity of variances was met, a one-way analysis of variance (ANOVA) was performed to compare differences among the experimental groups, followed by Tukey’s honestly significant difference (HSD) test for post hoc analyses. In cases where this assumption was not fulfilled, Welch’s ANOVA followed by the Games–Howell procedure was applied instead. Paw withdrawal thresholds values are expressed as mean ± SEM. The normality of data distribution was evaluated using the Shapiro–Wilk test. For within-group comparisons, two-tailed paired *t*-tests were applied, and effect sizes were calculated as Cohen’s dz. For between-group analyses, the assumption of homogeneity of variances was tested using Levene’s test. As this assumption was satisfied, one-way ANOVA was conducted, followed by Tukey’s HSD post hoc test to determine pairwise group differences. The histopathological results are presented as median values along with their corresponding minimum and maximum ranges. Groupwise differences were assessed with the nonparametric Mann–Whitney U test; all comparisons were two-tailed. A *p*-value of <0.05 was considered statistically significant.

## 3. Results

### 3.1. Biochemical Findings

#### 3.1.1. Analysis of MDA and tGSH Levels in Sciatic Nerve Tissue

**Figure 1:**
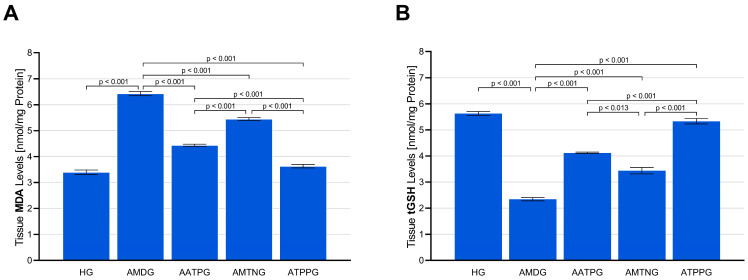
Effects of ATP, melatonin, TPP, and amiodarone administration on (**A**) MDA and (**B**) tGSH levels in rat sciatic nerve tissue. All data are presented as mean ± SEM (standard error of the mean).

As shown in Figure 1A and Table 2, the level of MDA in sciatic nerve tissue was significantly elevated in the amiodarone-only group (AMDG; 6.43 ± 0.08; vs. HG, *p* < 0.001) compared with the healthy control group (HG; 3.40 ± 0.09). Pre-administration of ATP (AATPG; 4.44 ± 0.04) effectively counteracted the amiodarone-induced rise in MDA levels and exerted stronger antioxidant activity than melatonin (AMTNG; 5.45 ± 0.06; vs. AMDG, *p* < 0.001; vs. AATPG, *p* < 0.001). Among all treatment strategies, TPP produced the most substantial suppression of MDA accumulation (ATPPG; 3.63 ± 0.07; vs. AMDG, *p* < 0.001), demonstrating significantly greater efficacy than both ATP and melatonin (ATPPG vs. AATPG and AMTNG, *p* < 0.001).

Administration of amiodarone markedly decreased tGSH levels in sciatic nerve tissue (AMDG; 2.34 ± 0.06) compared with the healthy control group (HG; 5.63 ± 0.08), and this reduction was statistically significant (*p* < 0.001). Pretreatment with ATP (AATPG; 4.12 ± 0.03), melatonin (AMTNG; 3.44 ± 0.13), or TPP (ATPPG; 5.33 ± 0.10) significantly prevented the amiodarone-induced depletion of tGSH (all vs. AMDG, *p* < 0.001). Among these interventions, TPP demonstrated the most pronounced protective effect (ATPPG vs. AATPG and AMTNG, *p* < 0.001), followed by ATP (AATPG vs. AMTNG, *p* = 0.013), whereas melatonin exerted the weakest inhibitory effect (Figure 1B and Table 2).

#### 3.1.2. Analysis of SOD and CAT Activities in Sciatic Nerve Tissue

**Figure 2:**
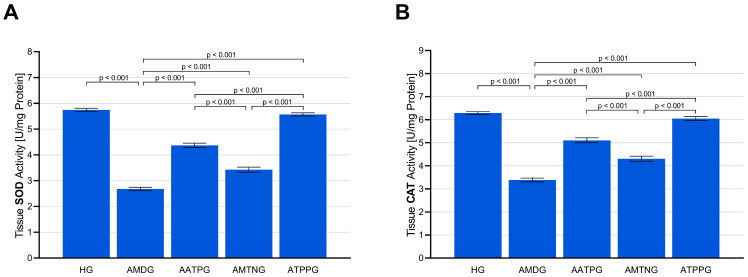
Impact of ATP, melatonin, TPP, and amiodarone administration on (**A**) SOD and (**B**) CAT activities in rat sciatic nerve tissue. All data are presented as mean ± SEM (standard error of the mean).

As presented in Figure 2A and Table 2, exposure to amiodarone led to a substantial decline in SOD activity within sciatic nerve tissue (AMDG; 2.68 ± 0.07; vs. HG, *p* < 0.001), showing a highly significant difference compared with the healthy control group (HG; 5.74 ± 0.06). Prophylactic administration of ATP (AATPG; 4.37 ± 0.09), melatonin (AMTNG; 3.43 ± 0.10), or TPP (ATPPG; 5.57 ± 0.06) effectively counteracted the enzyme activity loss induced by amiodarone, resulting in significantly higher SOD activity compared with the untreated group (all vs. AMDG, *p* < 0.001). Among these treatment strategies, TPP provided the most pronounced preservation of antioxidant capacity (ATPPG vs. AATPG and AMTNG, *p* < 0.001), while ATP exhibited an intermediate restorative effect (AATPG vs. AMTNG, *p* < 0.001) and melatonin exerted the weakest corrective influence.

Amiodarone exposure caused a pronounced reduction in CAT activity in sciatic nerve tissue (AMDG; 3.38 ± 0.08; vs. HG, *p* < 0.001), with values significantly lower than those of the healthy control group (HG; 6.29 ± 0.06). Pre-administration of ATP (AATPG; 5.11 ± 0.11), melatonin (AMTNG; 4.31 ± 0.11), or TPP (ATPPG; 6.05 ± 0.09) substantially prevented this enzyme activity deficit, leading to significantly enhanced CAT activity compared with the amiodarone-only group (all vs. AMDG, *p* < 0.001). Among the therapeutic agents evaluated, TPP displayed the strongest ability to maintain enzymatic defense (ATPPG vs. AATPG and AMTNG, *p* < 0.001), followed by ATP, which achieved a moderate improvement (AATPG vs. AMTNG, *p* < 0.001), whereas melatonin exerted the least protective action (Figure 2B and Table 2).

#### 3.1.3. Analysis of TNF-α, IL-1β, and IL-6 Levels in Sciatic Nerve Tissue

**Figure 3:**
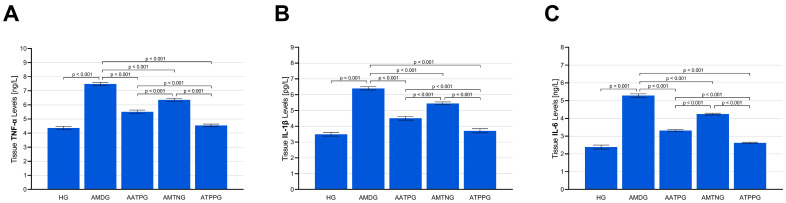
Effects of ATP, melatonin, TPP, and amiodarone administration on (**A**) TNF-α, (**B**) IL-1β and (**C**) IL-6 levels in rat sciatic nerve tissue. All data are presented as mean ± SEM (standard error of the mean).

As indicated by Figure 3A–C and Table 2, levels of the proinflammatory cytokines TNF-α, IL-1β, and IL-6 in sciatic nerve tissue were significantly elevated in animals treated with amiodarone alone (AMDG: TNF-α, 7.48 ± 0.12; IL-1β, 6.40 ± 0.10; IL-6, 5.29 ± 0.09) compared with the healthy control group (HG: TNF-α, 4.37 ± 0.11; IL-1β, 3.49 ± 0.11; IL-6, 2.39 ± 0.11) (all *p* < 0.001). Pretreatment with ATP (AATPG: TNF-α, 5.51 ± 0.12; IL-1β, 4.51 ± 0.12; IL-6, 3.32 ± 0.05), melatonin (AMTNG: TNF-α, 6.36 ± 0.11; IL-1β, 5.45 ± 0.09; IL-6, 4.24 ± 0.05), or TPP (ATPPG: TNF-α, 4.55 ± 0.09; IL-1β, 3.71 ± 0.13; IL-6, 2.62 ± 0.04) markedly prevented the cytokine elevations induced by amiodarone (all vs. AMDG, *p* < 0.001). Significant differences in TNF-α, IL-1β, and IL-6 levels were observed among all treatment groups (*p* < 0.001 for all comparisons). Among the tested agents, TPP produced the most substantial suppression of cytokine overproduction (ATPPG vs. AATPG and AMTNG, *p* < 0.001 for all parameters). In contrast, ATP demonstrated a moderate inhibitory capacity (AATPG vs. AMTNG, *p* < 0.001 for all parameters), whereas melatonin exhibited the least pronounced regulatory effect.

### 3.2. Paw Withdrawal Thresholds Findings

Baseline paw withdrawal thresholds did not differ significantly among the experimental groups. Following treatment, a marked reduction in paw withdrawal thresholds was observed in the amiodarone-only administered group (AMDG), indicating the development of neuropathic pain. In contrast, pretreatment with ATP, melatonin, or TPP significantly attenuated this decrease, demonstrating a protective analgesic effect.

**Figure 4:**
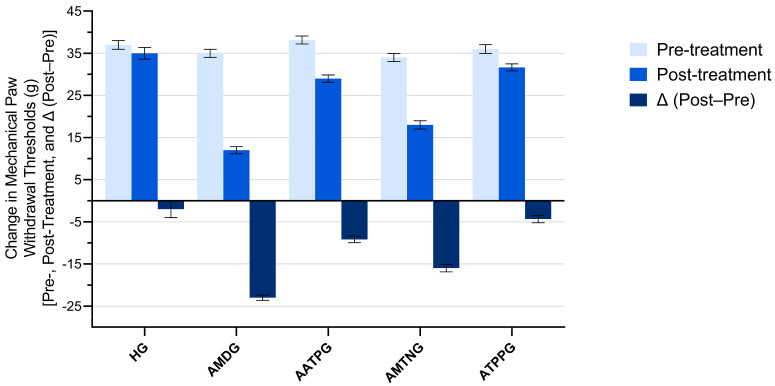
Comparison of pre- and post-treatment mechanical paw withdrawal thresholds within each group. All data are presented as mean ± SEM (standard error of the mean).

Within-group analyses revealed that AMDG exhibited the most pronounced decrease in paw withdrawal thresholds (Δ (post-treatment − pre-treatment) = −23.00 ± 0.63, t = −36.37, *p* < 0.001, dz = −14.846). Significant reductions were also observed in AMTNG (Δ = −16.00 ± 0.89, *p* < 0.001, dz = −7.303) and AATPG (Δ = −9.17 ± 0.79, *p* < 0.001, dz = −4.723), whereas ATPPG showed a moderate reduction (Δ = −4.33 ± 0.88, *p* = 0.004, dz = −2.006). No significant pre–post treatment difference was detected in the healthy control group (HG) (*p* = 0.367) (Figure 4 and Table 3).

**Figure 5:**
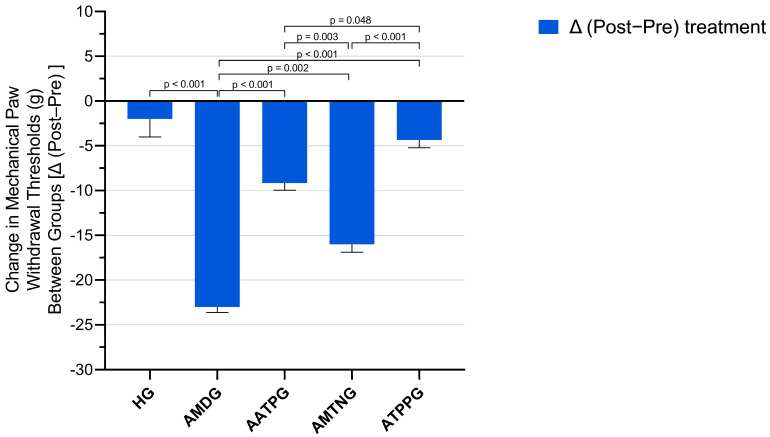
Between-group comparison of treatment-related changes in mechanical paw withdrawal thresholds (Δ post−pre). All data are presented as mean ± SEM (standard error of the mean).

Between-group comparisons of Δ values demonstrated a significant overall treatment effect (Levene’s F = 1.305, *p* = 0.295; ANOVA F (4,25) = 55.783, *p* < 0.001). Post hoc Tukey analysis showed that AMDG had significantly lower paw withdrawal thresholds than HG group (*p* < 0.001). Compared with AMDG, all cotreatment groups exhibited significantly higher paw withdrawal thresholds, indicating their ability to mitigate amiodarone-induced hyperalgesia to varying degrees (AATPG and ATPPG vs. AMDG, *p* < 0.001; AMTNG vs. AMDG, *p* = 0.002). AMTNG and AATPG differed significantly from both ATPPG (AMTNG vs. ATPPG, *p* < 0.001; AATPG vs. ATPPG, *p* = 0.048) and HG (AMTNG vs. HG, *p* < 0.001; AATPG vs. HG, *p* = 0.002), whereas no significant difference was observed between ATPPG and HG (*p* = 0.616) (Figure 5 and Table 4).

### 3.3. Histopathological Findings

**Figure 6:**
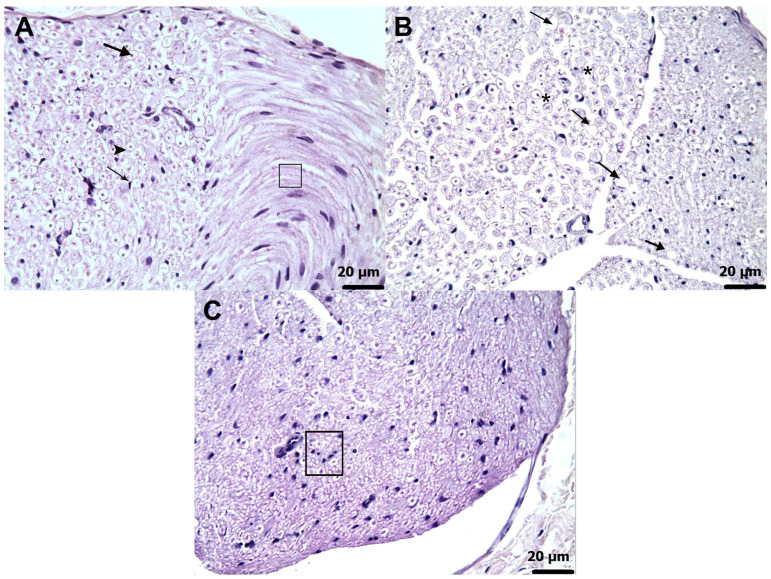
Micrographs of sciatic nerve tissue in rats. (**A**) HG group displaying normal structural organization with preserved axons (arrow), myelin sheath (arrowhead), Schwann cells (thin arrow), and nerve fibers (□) (H&E, ×40). (**B**) AMDG group exhibiting most severe axonal degeneration (arrows) accompanied by marked interstitial edema (*) (H&E, ×40). (**C**) AMDG group showing extensive proliferation of Schwann cells (□) (H&E, ×40). Pronounced histopathological alterations were observed in the damaged group compared with the preserved architecture in the healthy group.

**Figure 7:**
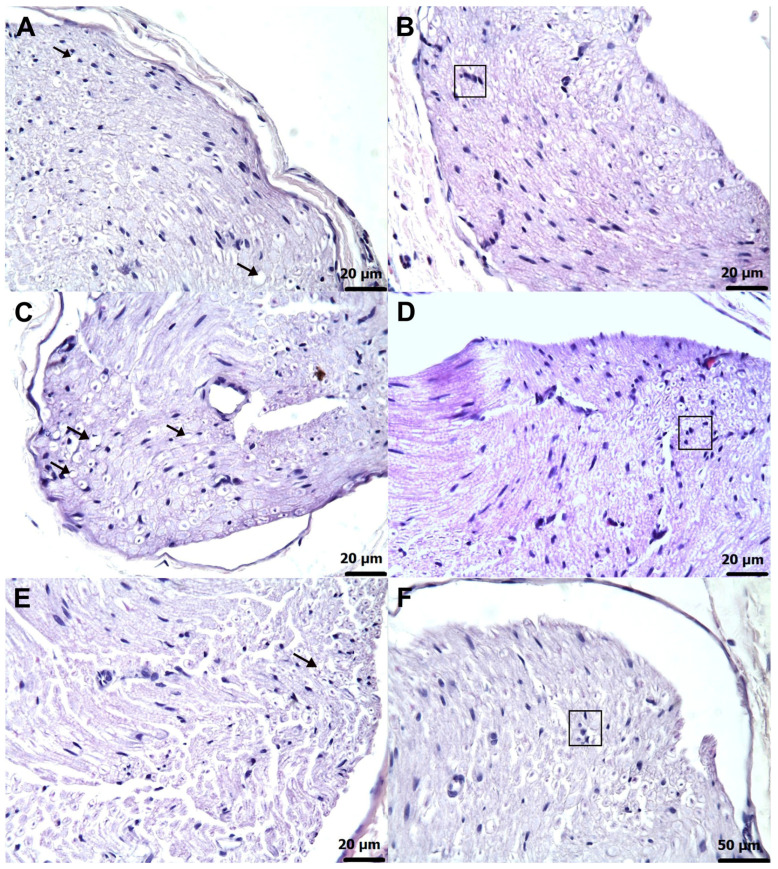
Micrographs of sciatic nerve tissue in rats. (**A**) AATPG group exhibiting moderate axonal degeneration (arrows) (H&E, ×40). (**B**) AATPG group showing moderate proliferation of Schwann cells (□) (H&E, ×40). (**C**) AMTNG group displaying severe axonal degeneration (arrows) (H&E, ×40). (**D**) AMTNG group demonstrating severe proliferation of Schwann cells (□) (H&E, ×40). (**E**) ATTPG group presenting mild axonal degeneration (arrow) (H&E, ×40). (**F**) ATTPG group exhibiting mild proliferation of Schwann cells (□) (H&E, ×40). The severity and extent of histopathological alterations varied among the treatment groups.

Microscopic examination revealed no evidence of structural abnormalities in the sciatic nerve tissue of healthy control animals (Figure 6A). In contrast, specimens obtained from animals treated with amiodarone alone (AMDG) exhibited severe axonal degeneration (grade 4) accompanied by extensive interstitial edema (grade 4) (Figure 6B). Furthermore, a marked proliferation of Schwann cells (grade 4) was clearly evident in this group (Figure 6C). In the AATPG group, histological assessment identified moderate axonal degeneration (grade 2) (Figure 7A) along with a grade 2 proliferative response of Schwann cells (Figure 7B). Nerve sections from animals in the AMTNG group displayed pronounced degenerative alterations in axons (grade 3) (Figure 7C), together with a grade 3 increase in Schwann cell proliferation (Figure 7D). In contrast, tissue samples from the ATPPG group exhibited only mild axonal degeneration (grade 1) (Figure 7E), and Schwann cell proliferation remained similarly low (grade 1) (Figure 7F). A semi-quantitative analysis of these histopathological alterations in rat sciatic nerve tissue, including intergroup statistical comparisons and associated *p*-values, is presented in Table 5.

## 4. Discussion

In the present study, the protective effects of ATP, melatonin, and TPP against amiodarone-induced neuropathy and neuropathic pain in rats were investigated through both biochemical and histopathological analyses and comparatively evaluated. Our findings indicate that amiodarone induces significant alterations in the biochemical composition and histoarchitectural organization of sciatic nerve tissue. As is well established, amiodarone inhibits mitochondrial complex I activity and induces uncoupled oxidative phosphorylation, thereby leading to a reduction in intracellular ATP levels [3]. With prolonged administration, this condition may ultimately result in the development of peripheral neuropathy [37,38]. In peripheral neuropathy, as nerve cells become progressively damaged, pain may be perceived even in regions that are normally insensitive to painful stimuli [39]. As is well established, pain constitutes the principal clinical manifestation of neuropathy [40]. The literature also emphasizes that amiodarone can lead to peripheral neuropathy, which manifests as pain and burning sensations in the hands and feet, and that long-term use may result in demyelination and axonal degeneration [41,42]. El-Bahri et al. described amiodarone-induced neuromyopathy as a condition causing pain and muscle weakness [7]. In our study, amiodarone-induced peripheral neuropathy was demonstrated by paw withdrawal thresholds measurements together with biochemical and histopathological findings. Consistent with our findings, previous studies have reported that amiodarone administered once daily at 50 mg/kg for 14 days induces the development of neuropathy [34]. Impairments in ATP production can exacerbate pain in peripheral neuropathies, and improving these mechanisms has therapeutic potential [12]. In our study, ATP significantly prevented the reduction in paw withdrawal thresholds, consistent with the literature.

Another molecule that reduced amiodarone-induced neuropathic pain in our study was melatonin. Prior studies also report that melatonin is effective in reducing neuropathic pain [19]. However, our findings indicate that melatonin was less effective than ATP in preventing amiodarone-related neuropathic pain. In a recent study we conducted, it was found that melatonin was less effective than ATP in protecting optic nerve tissue against 5-fluorouracil-induced oxidative and inflammatory damage [32]. Furthermore, TPP exhibited a stronger analgesic effect than both ATP and melatonin in amiodarone-induced neuropathic pain, consistent with reports emphasizing TPP’s efficacy in drug-induced neuropathic pain [27].

Enhanced production of ROS, along with impaired antioxidant defense mechanisms, plays a pivotal role in the pathogenesis of amiodarone-induced toxicity [43]. In sciatic nerves from amiodarone-treated animals, MDA levels were significantly higher than in the healthy, ATP, melatonin, and TPP groups. MDA, used in assessing amiodarone neurotoxicity, is a biomarker of oxidative stress and is associated with neurodegenerative diseases [44]. Peripheral neuropathic pain is associated with mitochondrial dysfunction and decreased ATP [11,12]. Fernyhough et al. reported that oxidative stress and ATP depletion lead to neuropathy [45]. In line with the literature [46], our findings show that ATP may alleviate nerve injury by reducing oxidative stress. Experimental evidence demonstrating that melatonin, an antioxidant and anti-inflammatory molecule [47], reduces MDA levels and lipid peroxidation (LPO) in diabetic neuropathy is consistent with our findings [48]. However, ATP more effectively attenuated the increase in MDA levels in nerve tissue compared with melatonin. These findings are in line with previous evidence demonstrating that ATP provides stronger protection than melatonin against 5-fluorouracil-induced oxidative and inflammatory damage in optic nerve tissue [32]. Moreover, TPP exhibited the most pronounced protective effect against the amiodarone-induced elevation in MDA levels in sciatic nerve tissue. Our findings are supported by studies demonstrating TPP’s efficacy in DIPN [27,49].

The inhibition of antioxidant defense mechanisms represents a crucial factor in the pathogenesis of amiodarone-induced toxicity [10]. tGSH, SOD, and CAT are key components of the cellular antioxidant defense machinery, playing a crucial role in protecting cells from oxidative damage [50]. In our study, the levels of tGSH, SOD, and CAT were significantly decreased in the amiodarone group compared with the control group. Our results parallel reports that amiodarone suppresses antioxidant expression and increases oxidative damage [43]. Experimental and preclinical data suggest that antioxidant agents may reduce amiodarone-induced oxidative damage [51,52]. Our findings are in agreement with previous reports [46], indicating that ATP exerts a notable antioxidative effect by preventing the decline in antioxidant markers such as tGSH, SOD, and CAT. The finding that melatonin contributed to the maintenance of antioxidant balance by preventing reductions in GSH, SOD, and CAT levels is in agreement with previous literature [53]. However, in the present study, the antioxidant activity of melatonin was less pronounced than that of ATP. These findings are in line with previous studies reporting that ATP exerts a greater protective effect than melatonin in drug-induced oxidative stress [32]. Furthermore, in the present study, TPP more significantly attenuated the amiodarone-induced disruption of the oxidant/antioxidant balance in favor of oxidants in nerve tissue compared with ATP and melatonin. The antioxidant action of TPP is well established [54], and studies showing that TPP prevents decreases in enzymatic and non-enzymatic antioxidants (tGSH, SOD, CAT) support our experimental results [55].

To further elucidate the mechanisms contributing to amiodarone-induced neuropathy and the development of neuropathic pain, pro-inflammatory cytokines including TNF-α, IL-1β, and IL-6 were also quantified in nerve tissue. In neuropathies, immune-cell accumulation leads to the release of inflammatory cytokines; serum TNF and IL-6 expression increase during this process [56]. In our study, inflammatory cytokine levels were elevated in the nerve tissue of amiodarone-treated animals, which is consistent with previous reports demonstrating amiodarone-induced tissue injury [57,58]. While we found no studies specifically investigating the effects of ATP on inflammatory cytokines in sciatic nerve tissue, a study conducted in liver tissue reported similar findings, showing that maintaining ATP levels prevented increases in TNF-α, IL-1β, and IL-6 [59]. The observation that melatonin contributed to the maintenance of inflammatory cytokine homeostasis by preventing elevations in TNF-α, IL-1β, and IL-6 levels is in agreement with previous reports [48]. However, in the present study, the ability of melatonin to preserve inflammatory cytokine levels was less pronounced than that of ATP. These findings are in line with previous reports demonstrating that ATP exerts a greater protective effect than melatonin against drug-induced inflammatory injury [32]. Our findings demonstrated that TPP exerted the most pronounced effect in preventing elevations in pro-inflammatory cytokines, in agreement with previous reports indicating that TPP helps maintain cytokine homeostasis in DIPN [27].

The histopathological evidence further substantiated the biochemical results, reinforcing the overall interpretation of our data. The histopathological changes observed in the sciatic nerves, including axonal degeneration, proliferation of Schwann cells, and interstitial edema, in animals treated with amiodarone are in agreement with previous reports in the literature [60,61]. Niimi et al. concluded that this phenomenon may lead to demyelination in the peripheral nervous system, associated with impaired lysosomal degradation in Schwann cells [62]. Supporting our findings, studies in DIPN have shown reduced neuronal injury, demyelination, and leukocyte infiltration following agents with potential neuroprotective effects [27,49]. In our study, ATP administration ameliorated histopathological alterations in sciatic nerve tissue, in agreement with previous studies reporting that reduced ATP production contributes to morphological changes associated with peripheral neuropathy [63]. Melatonin also led to improvements in histopathological alterations observed in the sciatic nerve tissue in our study, and this finding is consistent with data reported in the literature [19]. However, in the present study, the ability of melatonin to preserve histopathological architecture was less pronounced than that of ATP. Our findings are in line with previous reports demonstrating that ATP affords greater histopathological protection against drug-induced optic nerve injury compared with melatonin [32]. In our study, TPP, tested for its effect against amiodarone-induced neuropathic pain, provided better histopathological protection than both ATP and melatonin, consistent with the previous literature [27].

## 5. Conclusions

Amiodarone administration led to a significant elevation in oxidative stress biomarkers and pro-inflammatory cytokines within sciatic nerve tissue, accompanied by a marked reduction in antioxidant defense markers and paw withdrawal thresholds. These biochemical alterations were corroborated by corresponding histopathological findings. Among the agents evaluated for their neuroprotective potential, TPP exhibited the most pronounced protective effect, followed by ATP and melatonin. Collectively, our biochemical and histopathological data indicate that prolonged amiodarone exposure may contribute to the development of neuropathic pain, and that TPP could offer superior neuroprotective efficacy compared with ATP and melatonin. However, further clinical studies are required to confirm the translational relevance and therapeutic potential of these agents.

### Limitations

This study has several noteworthy limitations. First, ATP, melatonin and TPP were evaluated only as single-agent interventions. The absence of dual or triple combination groups restricts the ability to determine whether these agents might exert synergistic, additive, or antagonistic interactions when administered concurrently. Future research incorporating combinatorial treatment paradigms is needed to clarify whether multi-targeted strategies could further enhance neuroprotection against amiodarone-induced injury. Second, only male rats were included in the experimental design. Although this approach reduces biological variability and aligns with many preclinical neuropathy models, it limits the evaluation of potential sex-related differences in susceptibility to amiodarone-induced neuropathy and in the therapeutic responsiveness to ATP, melatonin or TPP. Given the documented influence of sex on pain perception, mitochondrial regulation, and inflammatory signaling, future studies should incorporate both sexes to determine whether treatment outcomes diverge between male and female subjects. Third, although the doses of ATP, melatonin and TPP were selected based on prior studies demonstrating reliable antioxidant and anti-inflammatory efficacy, the lack of dose–response assessments limits the interpretation of their minimal effective doses, maximal therapeutic thresholds, and overall safety–efficacy profiles. Systematic dose-ranging studies are required to establish optimized dosing regimens and to determine whether lower or higher doses may produce superior pharmacodynamic or neuroprotective outcomes. Fourth, more advanced and quantitatively oriented histopathological assessments, including immunohistochemical detection of S100 calcium binding protein β and morphometric analyses of axonal diameter and myelin thickness, were not undertaken. Incorporation of such methodologies would have yielded a more nuanced and structurally detailed characterization of sciatic nerve pathology and is expected to substantially enhance the interpretive depth of histopathological outcomes in future investigations. Taken together, these limitations underscore the need for subsequent investigations incorporating combination treatment paradigms, sex inclusive experimental designs, systematic dose response analyses and advanced histopathological methodologies such as immunohistochemical markers and quantitative morphometric assessments in order to more comprehensively delineate the therapeutic relevance and translational potential of ATP, melatonin and TPP in amiodarone-induced neuropathy.

## Figures and Tables

**Table 1:** Semi-quantitative grading criteria for sciatic nerve injury.

Grade	Axonal Degeneration	Schwann Cell Proliferation (Focal Clusters of Adjacent Cells)	Interstitial Edema (% of Tissue Area)
0	None	None	0%
1	1 axon	1 focal cluster	<5%
2	2–3 axons	2–3 focal clusters	6–15%
3	4–5 axons	4–5 focal clusters	16–25%
4	≥6 axons	≥6 focal clusters	≥26%

Six representative microscopic fields were assessed per group at 40× magnification.

**Table 2:** Comparative analysis of the effects of ATP, melatonin, TPP, and amiodarone on oxidative, antioxidative, and pro-inflammatory parameters in rat sciatic nerve.

	Post hoc Test *p*-Values						
Group Comparisons	MDA *	tGSH **	SOD *	CAT *	TNF-α *	IL-1β *	IL-6 **
HG vs. AMDG	<0.001	<0.001	<0.001	<0.001	<0.001	<0.001	<0.001
HG vs. AATPG	<0.001	<0.001	<0.001	<0.001	<0.001	<0.001	<0.001
HG vs. AMTNG	<0.001	<0.001	<0.001	<0.001	<0.001	<0.001	<0.001
HG vs. ATPPG	0.149	0.242	0.512	0.360	0.772	0.643	0.349
AMDG vs. AATPG	<0.001	<0.001	<0.001	<0.001	<0.001	<0.001	<0.001
AMDG vs. AMTNG	<0.001	<0.001	<0.001	<0.001	<0.001	<0.001	<0.001
AMDG vs. ATPPG	<0.001	<0.001	<0.001	<0.001	<0.001	<0.001	<0.001
AATPG vs. AMTNG	<0.001	0.013	<0.001	<0.001	<0.001	<0.001	<0.001
AATPG vs. ATPPG	<0.001	<0.001	<0.001	<0.001	<0.001	<0.001	<0.001
AMTNG vs. ATPPG	<0.001	<0.001	<0.001	<0.001	<0.001	<0.001	<0.001
F value	346.018	286.043 ^a^	295.187	174.922	135.431	118.787	241.618 ^a^
df (df1/df2)	4/25	4/11.377 ^b^	4/25	4/25	4/25	4/25	4/12.223 ^b^
*p*	<0.001	<0.001	<0.001	<0.001	<0.001	<0.001	<0.001

* One-way ANOVA was performed to examine intergroup differences, followed by Tukey’s honestly significant difference (HSD) test for post hoc pairwise comparisons. ** Welch’s ANOVA was applied to evaluate group differences, with the Games–Howell procedure used for subsequent post hoc analyses. ^a^ means asymptotically F distributed. ^b^ means Welch ANOVA df. For all groups *n* = 6.

**Table 3:** Within-group comparison of mechanical paw withdrawal thresholds before and after treatment.

		Paw Withdrawal Thresholds (g)							
Groups	*n*	Pre-Treatment	Post-Treatment	Δ (Post−Pre)	Shapiro–Wilk Pre-Treatment *p* Values	Shapiro–Wilk Post-Treatment *p* Values	*t*	*p* (Two-Tailed)	Cohen’s dz
HG	6	37.00 ± 1.03	35.00 ± 1.37	−2.00 ± 2.02	0.757	0.886	−0.992	0.367	−0.405
AMDG	6	35.00 ± 0.97	12.00 ± 0.86	−23.00 ± 0.63	0.739	0.320	−36.366	<0.001	−14.846
AATPG	6	38.17 ± 0.95	29.00 ± 0.86	−9.17 ± 0.79	0.287	0.320	−11.569	<0.001	−4.723
AMTNG	6	34.00 ± 0.97	18.00 ± 0.97	−16.00 ± 0.89	0.739	0.739	−17.889	<0.001	−7.303
ATPPG	6	36.00 ± 1.07	31.67 ± 0.84	−4.33 ± 0.88	0.817	0.493	−4.914	0.004	−2.006

Values are expressed as mean ± SEM (standard error of the mean). Δ indicates the change (post − pre). Shapiro–Wilk test was used to assess normality for pre- and post-treatment values. Since *p* ≥ 0.05 indicated no significant deviation from normality, two-tailed paired *t*-tests were applied for within-group comparisons, and two-tailed *p* values are reported. Cohen’s dz values represent effect sizes. Statistical significance was set at *p* < 0.05.

**Table 4:** Between-group comparison of changes in mechanical paw withdrawal thresholds before and after treatment, and associated analgesic activities.

	Paw Withdrawal Thresholds (g)						
Groups	Pre-Treatment	Post-Treatment	Δ (Post−Pre)	Shapiro–Wilk Pre-Treatment *p*-Value	Shapiro–Wilk Post-Treatment *p*-Value	Shapiro–Wilk Δ (Post−Pre) *p*-Value	Analgesic Activity (%)
HG	37.00 ± 1.03	35.00 ± 1.37	−2.00 ± 2.02	0.757	0.886	0.864	91.30
AMDG	35.00 ± 0.97	12.00 ± 0.86	−23.00 ± 0.63	0.739	0.320	0.456	-
AATPG	38.17 ± 0.95	29.00 ± 0.86	−9.17 ± 0.79	0.287	0.320	0.452	60.13
AMTNG	34.00 ± 0.97	18.00 ± 0.97	−16.00 ± 0.89	0.739	0.739	0.783	30.43
ATPPG	36.00 ± 1.07	31.67 ± 0.84	−4.33 ± 0.88	0.817	0.493	0.405	81.17
Group comparisons	Pre-treatment *p* value	Post-treatment *p* value	Δ (post−pre) *p*-value				
HG vs. AMDG	0.621	<0.001	<0.001				
HG vs. AATPG	0.919	0.002	0.002				
HG vs. AMTNG	0.239	<0.001	<0.001				
HG vs. ATPPG	0.952	0.159	0.616				
AMDG vs. AATPG	0.195	<0.001	<0.001				
AMDG vs. AMTNG	0.952	0.002	0.002				
AMDG vs. ATPPG	0.952	<0.001	<0.001				
AATPG vs. AMTNG	0.048	<0.001	0.003				
AATPG vs. ATPPG	0.549	0.348	0.048				
AMTNG vs. ATPPG	0.621	<0.001	<0.001				
F	2.693	95.011	55.783				
df (df1/df2)	4/25	4/25	4/25				
significance	0.054	<0.001	<0.001				

Values are expressed as mean ± SEM. Δ values represent the change in paw withdrawal thresholds after and before treatment. Shapiro–Wilk test was used to assess normality for pre-treatment, post-treatment and Δ values. Since *p* ≥ 0.05 indicated no significant deviation from normality and Levene’s test (pre-treatment, *p* = 0.941; post-treatment, *p* = 0.497; Δ, *p* = 0.295) was not significant, one-way ANOVA followed by Tukey honestly significant difference (HSD) post hoc test was applied. For all groups *n* = 6.

**Table 5:** Semi-quantitative assessment of histopathological findings in rat sciatic nerve.

	Histopathological Grading Data		
Groups	Axonal Degeneration	Proliferation in Schwann Cells	Interstitial Edema
HG	0.00 (0.00–0.00)	0.00 (0.00–0.00)	0.00 (0.00–0.00)
AMDG	4.00 (3.00–4.00)	4.00 (3.00–4.00)	3.50 (3.00–4.00)
AATPG	2.00 (1.00–3.00)	2.00 (1.00–3.00)	0.00 (0.00–1.00)
AMTNG	2.50 (2.00–3.00)	3.00 (2.00–3.00)	0.00 (0.00–1.00)
ATPPG	1.00 (0.00–2.00)	1.00 (0.00–2.00)	0.00 (0.00–1.00)
Group comparisons	*p*-values		
HG vs. AMDG	0.002	0.001	0.002
HG vs. AATPG	0.002	0.002	0.138
HG vs. AMTNG	0.002	0.002	0.138
HG vs. ATPPG	0.021	0.006	0.317
AMDG vs. AATPG	0.007	0.003	0.003
AMDG vs. AMTNG	0.011	0.006	0.003
AMDG vs. ATPPG	0.003	0.002	0.002
AATPG vs. AMTNG	0.423	0.057	1.000
AATPG vs. ATPPG	0.020	0.067	0.523
AMTNG vs. ATPPG	0.006	0.004	0.523

Data are shown as median (min–max). Statistical analyses were performed using the Mann–Whitney U test. All groups consisted of six samples (*n* = 6).

## Data Availability

The original contributions presented in this study are included in this article/Supplementary Materials. Further inquiries can be directed to the corresponding author.

## Supplementary Materials

The following supporting information can be downloaded at: https://www.mdpi.com/article/10.3390/biomedicines13122965/s1, Table S1. Verification of the normal distribution assumption for biochemical markers in rat sciatic nerve tissue using the Shapiro–Wilk test; Table S2. Evaluation of variance homogeneity across the datasets for MDA, tGSH, SOD, CAT, TNF-α, IL-1β, and IL-6 parameters.

## Abbreviations

The following abbreviations are used in this manuscript:

HG	healthy group
AMDG	amiodarone alone group
AATPG	amiodarone + ATP group
AMTNG	amiodarone + melatonin group
ATPPG	amiodarone + TPP group
ATP	adenosine triphosphate
TPP	thiamine pyrophosphate
MDA	malondialdehyde
tGSH	total glutathione
SOD	superoxide dismutase
CAT	catalase
TNF-α	tumor necrosis factor-alpha
IL-1β	interleukin one beta
IL-6	interleukin six
Δ (post−pre)	Net change from baseline (post minus pre)
