# Supplementary material for: Comparative Investigation of the Effects of Adenosine Triphosphate, Melatonin, and Thiamine Pyrophosphate on Amiodarone-Induced Neuropathy and Neuropathic Pain in Male Rats
Source: Biomedicines. 2025 Dec 2;13(12):2965. doi: 10.3390/biomedicines13122965 (PMC12730428; doi:10.3390/biomedicines13122965)
Supplement: Supplementary file 1 [file biomedicines-13-02965-s001.zip › Table S1-R1.pdf]

Table S1. Verification of the normal distribution assumption for biochemical markers in rat sciatic nerve tissue using the Shapiro–Wilk test

			**Biochemical Variables**						
		**Shapiro-Wilk**	**MDA**	**tGSH**	**SOD**	**CAT**	**TNF-α**	**IL-1β**	**IL-6**
Groups	HG	statistic	0.925	0.879	0.938	0.962	0.866	0.951	0.838
		df	6	6	6	6	6	6	6
		sig.	0.543	0.263	0.641	0.836	0.212	0.749	0.125
	AMDG	statistic	0.840	0.975	0.953	0.903	0.869	0.979	0.978
		df	6	6	6	6	6	6	6
		sig.	0.132	0.922	0.766	0.394	0.222	0.949	0.941
	AATPG	statistic	0.986	0.911	0.941	0.909	0.949	0.843	0.935
		df	6	6	6	6	6	6	6
		sig.	0.977	0.445	0.666	0.427	0.730	0.139	0.619
	AMTNG	statistic	0.985	0.871	0.959	0.852	0.854	0.940	0.950
		df	6	6	6	6	6	6	6
		sig.	0.973	0.230	0.810	0.163	0.168	0.657	0.742
	ATPPG	statistic	0.918	0.975	0.994	0.954	0.976	0.969	0.892
		df	6	6	6	6	6	6	6
		sig.	0.491	0.924	0.997	0.775	0.930	0.888	0.331

Footnotes: As the datasets for MDA, tGSH, SOD, CAT, TNF-α, IL-1β, and IL-6 satisfied the assumption of normality, intergroup differences were analyzed using ANOVA. For all groups n = 6.

Abbreviations: ATP: adenosine triphosphate; TPP: thiamine pyrophosphate; HG: healthy group; AMDG: amiodarone alone group; AATPG: amiodarone + ATP group; AMTNG: amiodarone + melatonin group; ATPPG: amiodarone + TPP group; MDA: malondialdehyde; tGSH: total glutathione; SOD: superoxide dismutase; CAT: catalase; TNF-α: tumor necrosis factor-alpha; IL-1β: interleukin one beta; IL-6: interleukin six; df: degrees of freedom; sig: significance.
