# Supplementary material for: Comparative Investigation of the Effects of Adenosine Triphosphate, Melatonin, and Thiamine Pyrophosphate on Amiodarone-Induced Neuropathy and Neuropathic Pain in Male Rats
Source: Biomedicines. 2025 Dec 2;13(12):2965. doi: 10.3390/biomedicines13122965 (PMC12730428; doi:10.3390/biomedicines13122965)
Supplement: Supplementary file 1 [file biomedicines-13-02965-s001.zip › Table S2-R1.pdf]

Table S2. Evaluation of variance homogeneity across the datasets for MDA, tGSH, SOD, CAT, TNF-α, IL-1β, and IL-6 parameters

	**Biochemical Variables**						
	**MDA**	**tGSH**	**SOD**	**CAT**	**TNF-α**	**IL-1β**	**IL-6**
Levene's statistic	1.438	4.333	0.696	1.390	0.306	0.567	4.140
df1	4	4	4	4	4	4	4
df2	25	25	25	25	25	25	25
sig.	0.251	0.008	0.602	0.266	0.871	0.689	0.010

Footnotes: As the homogeneity of variances assumption was met, Tukey's honestly significant difference (HSD) test was applied for post hoc comparisons of MDA, SOD, CAT, TNF-α, and IL-1β levels. Conversely, because this assumption was violated, the Games–Howell test was performed for the post hoc analysis of tGSH and IL-6 data. For all groups n = 6.

Abbreviations: MDA: malondialdehyde; tGSH: total glutathione; SOD: superoxide dismutase; CAT: catalase; TNF-α: tumor necrosis factor-alpha; IL-1β: interleukin one beta; IL-6: interleukin six; df: degrees of freedom; sig: significance.
